# Precise dating of large flank collapses by single-grain ^40^Ar/^39^Ar on pyroclastic deposits from the example of Flores Island (Azores)

**DOI:** 10.1038/s41598-024-62583-1

**Published:** 2024-05-24

**Authors:** A. Hildenbrand, F. O. Marques, A. Pereira, S. Nomade, F. Hevia-Cruz

**Affiliations:** 1 Université Paris-Saclay, CNRS, GEOPS, 91405 Orsay, France; 2 Lisbon, Portugal; 3 LSCE, CEA, IPSL and Univ. Paris-Saclay, Gif-Sur-Yvette, France

**Keywords:** ^40^Ar/^39^Ar, Single-grain, Anorthoclase, Flank collapse, Blast, Differentiation, Inflation, Failure, Natural hazards, Solid Earth sciences

## Abstract

Large-scale flank collapses are one of the main hazards associated with the evolution of volcanic islands. Precisely dating such events is critical to evaluate the frequency of destabilization episodes and further assess the triggering mechanism(s) associated with internal and/or external factors, such as volcano dynamics, regional tectonics, and global paleoclimatic changes. Here, we constrain the age of a pumice-rich pyroclastic deposit exposed on the eastern flank of Flores Island (Azores), which we interpret as a co-blast deposit generated by a major flank collapse that destroyed the whole western flank of the former volcanic edifice. Twelve single-grain ^40^Ar/^39^Ar analyses, performed on 250–500 µm anorthoclase feldspars (mean K/Ca close to 5) with our high-sensitivity multi-collector NGX mass spectrometer, provide a robust weighted mean age of 1.32 ± 0.01 Ma for this eruption. This new age is consistent with previous K/Ar data bracketing the flank collapse between 1.30 ± 0.04 and 1.18 ± 0.09 Ma, and indicates that this event occurred at the end of the main construction phase of the volcano. The explosion produced pumice-rich layers preceded by a lahar as attested by a polygenetic mudflow deposit underlying the dated deposit. From the geochemistry of lavas erupted just before and after the collapse, we speculate upon the possible role of magmatic processes on flank destabilization. We propose a first hypothesis where differentiation in a shallow magma reservoir could have favored edifice inflation, ground shaking, and flank failure, triggering a decompression-induced violent eruption. Overall, our study shows that high-sensitivity mass spectrometers have now reached analytical performances allowing to measure precisely and accurately ages on relatively small and moderately K-rich single feldspars, which is of the utmost importance for dating heterogeneous blasts and tephra deposits that may have been induced by large-scale flank collapses during the late Quaternary.

## Introduction

Giant catastrophic flank collapses and resulting tsunamis represent a major threat for populations living on volcanic ocean islands and potentially distant coastlines. Over the last decades, tens of such collapses have been recognized worldwide. The most impressive cases were reported in Hawaii, where individual failure volumes may have reached thousands of cubic kilometers ^1–3^. Other giant landslides with volumes exceeding a hundred cubic kilometers have been also recognized, for example in the Canary Islands ^4–9^, in Polynesia ^10–12^ and Cape Verde ^13–16^. However, such events have been shown to happen quite rarely, with typical recurrence on the order of a few tens to hundreds of thousand years ^17^. Smaller volcanic islands may be prone to less voluminous but more frequent destabilization episodes, representing an underestimated source of hazards and risk (e.g., Refs.^18–21^) for nearby islands. Precisely dating past events is critical to evaluate the frequency of destabilization episodes and assess their triggering mechanism(s).

In most cases, the timing of large catastrophic landslides is bracketed by radiometric dating of (1) the pre-collapse volcanic units cut by the landslide scar, and (2) the post-collapse volcanic activity (e.g., Refs.^6,8,9,11,12,14,22–26^). While such an approach has given tight temporal constraints on a few voluminous flank collapses (> 10 km^3^), many destabilization events remain poorly dated. In very few cases, ^40^Ar/^39^Ar dating has been achieved on individual crystals from blast deposits associated with a given landslide, e.g., the Icod slide in Tenerife ^27^. Single-grain ^40^Ar/^39^Ar laser-fusion dating on individual feldspars and/or feldspathoids has increasingly proved to be very useful to precisely date Quaternary tephra deposits in various geodynamic settings (e.g., Refs.^28–32^). However, such analyses are often achieved on K-rich minerals such as sanidine and/or leucite.

Here we show that high-sensitivity multi-collector mass spectrometers such as the NGX600 (Isotopx) featuring full array of ATONA amplifiers have now reached analytical performances enabling precise single-grain age determination on moderately sized anorthoclase feldspars (250–500 µm) with modest Potassium content, as exemplified by the current work on Flores Island (Azores). We then discuss the possible influence of various factors on the onset of lateral flank destabilization. The obtained new age, complemented with new whole-rock geochemical analyses on pre- and post-collapse lava flows dated by unspiked K/Ar on groundmass (Ref.^21^ and this study), allows us to conduct educated speculation on the possible links between magma storage and evolution, edifice inflation and flank failure. For the sake of comparison, all the ages considered here are quoted at the 2σ confidence level.

## Geological and geodynamic background

**Figure 1:**
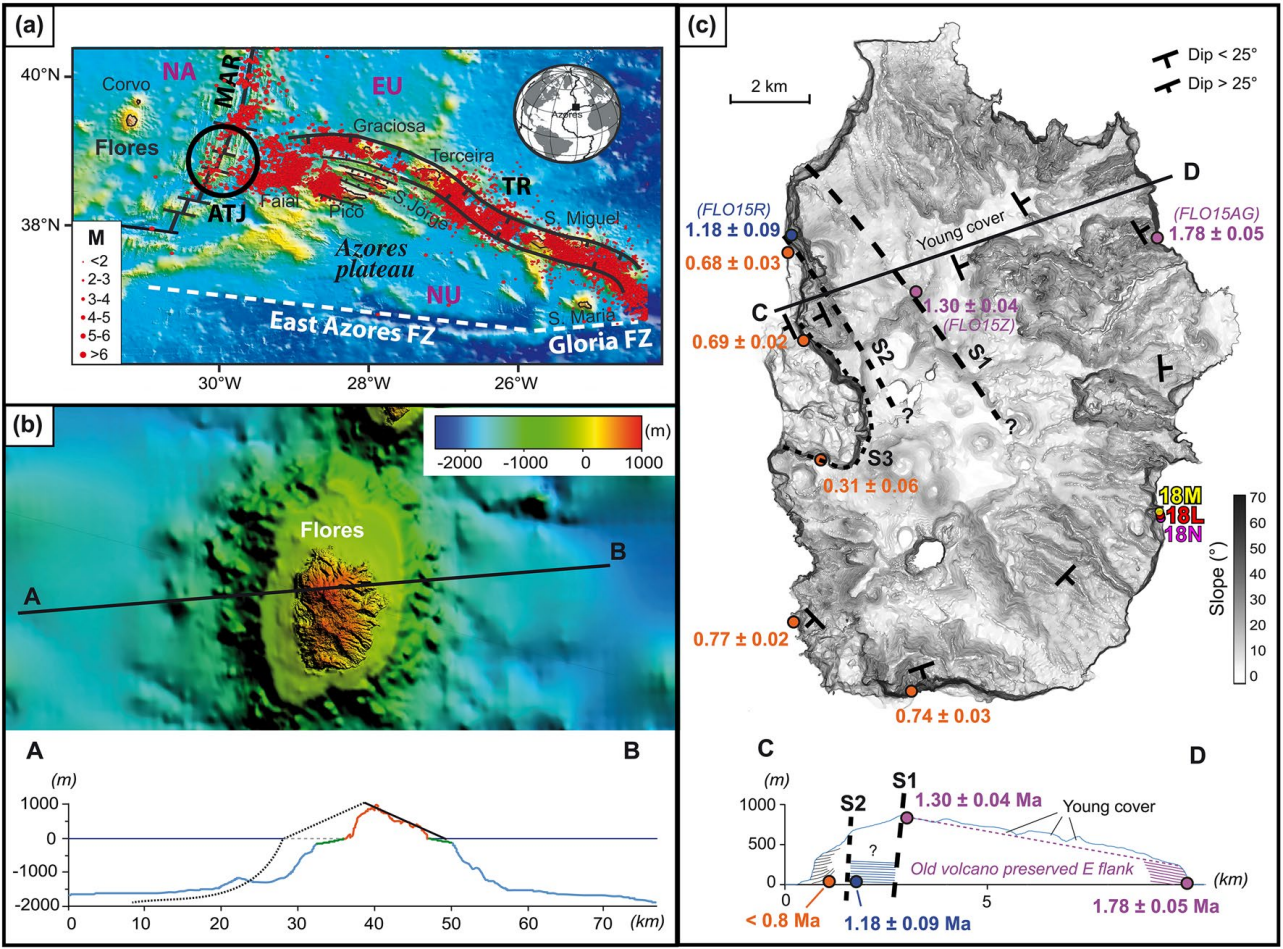
(**a**) Location of Flores Island on the North American plate (NA). Black lines show the axis of the MAR and associated fracture zones. Ticked lines bound the main horst and graben structures making up the eastern branch of the Azores triple junction (ATJ), where present deformation between Eurasia (EU) and Nubia (NU) is diffuse ^33,34^. Seismicity over the period 1981–2011 is shown by red dots; *M* magnitude. *TR* Terceira Rift, *EAFZ* East Azores Fracture Zone. (**b**) DEM and cross-section showing the full morphology of Flores Island, from EMODnet public data. Red and blue colors on the profile show the topography above and below sea level, respectively; green color highlights the extent of shallow present insular shelves created by recent marine erosion. The black solid line shows the main island's eastern slope, whereas the black dotted line shows the expected full western slope in the hypothesis of a symmetric volcanic edifice. (**c**) Shaded relief map (illumination from the top) of the island (source Hildenbrand et al. ^21^). The trace of the inferred scars is drawn with black dashed lines. The geometry of the various units and selected K/Ar ages (in Ma) are shown on the map and synthetic cross-section. The position of samples FLO15R, Z and AG and our new samples (18L, 18M and 18N) is shown. All images in the various panels are after ^21^, modified.

Flores Island, in the western Azores, is a relatively small volcanic island edified mainly during the Quaternary on the western flank of the Mid-Atlantic Ridge (MAR), close to the Azores Triple Junction (ATJ) between the North American, Eurasian and Nubian lithospheric plates (Fig. 1a). While the eastern and central Azores islands have been strongly influenced by regional tectonic deformation along the eastern branch of the TJ (e.g., Refs.^33,34^), Flores is located in a relatively quiet tectonic zone, with no current seismicity. The volcanic evolution of Flores seems mostly driven by decompression melting of an anomalously hot or volatile-enriched mantle (e.g., Refs.^35,36^), with no significant influence of lithospheric deformation.

Recent geomorphological, geological and geochronological analyses by our research group show that the Flores volcanic edifice is significantly asymmetric (Fig. 1b). Half of the island is indeed missing, whereas a bathymetric high is observed tens of kilometers offshore to the west, despite a deeper expected seafloor due to the age of the oceanic lithosphere (higher expected thermal subsidence). In 2018, Hildenbrand and collaborators ^21^ hypothesized that these observations were the result of major repeated destruction by west-directed flank collapses and related accumulation of large debris avalanches far beyond the submarine slope of the island. Onshore morphological, geological and geochronological data further show that the present island comprises nested volcanic successions with a westward age decrease, separated by major scars. Such geometric imbrication points to repeated westward flank destabilization interspersed with successive volcano reconstruction during the late Quaternary. The current western coast is being affected by a ca. 2 km-wide U-shape structure (S3, Fig. 1c) generated by ongoing outward flank creeping. Recent movement along this structure may have produced shallow slides and fissure eruptions during Holocene times ^37^. Older albeit much more voluminous and catastrophic destabilization episodes have been further evidenced ^21^. A first major flank collapse cut the full western flank of the former shield between 1.30 ± 0.04 Ma and 1.18 ± 0.09 Ma, producing a scarp partly visible in the northwestern half of the island (S1, Fig. 1c). After partial concealing by subsequent volcanic activity, a second large flank collapse between 1.18 ± 0.09 Ma and 0.77 ± 0.02 Ma removed part of the reconstructed volcano. The trace of this second main destabilization episode is partly visible as a slightly arcuate scar (S2, Fig. 1c), the southern extent of which was extensively concealed by more recent volcanic products. Yet, the exact volume and age of each landslide, especially the most voluminous (S1), remains poorly constrained, precluding any robust evaluation of the causes and mechanisms of flank destabilization.

## Discovery of pumice-rich deposits

**Figure 2:**
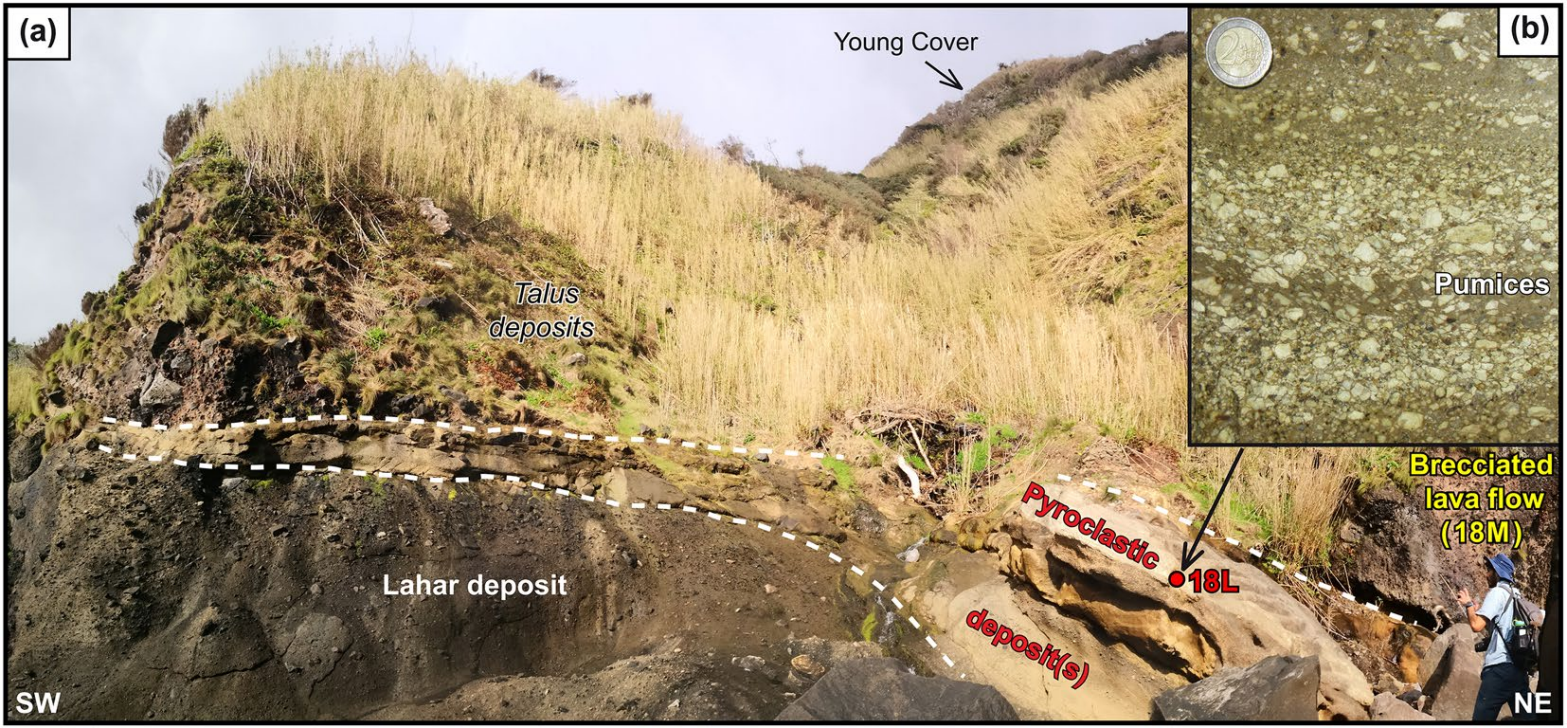
Field relationships observed on the SE coast of Flores (Photo taken by A. Hildenbrand).

As the western side of Flores comprises recent volcanic units, any potential deposit associated with an old flank collapse may only occur on the preserved eastern flank of the former volcano. With this in mind, we concentrated our new investigations along the eastern coast of the island. We systematically avoided the sectors that have been widely covered by young volcanic units and/or affected by recent stream erosion, and targeted accessible coastal segments preserved from major sea-wave erosion to get closer to the outer (upper) flank of the older volcano. The old volcanic succession could be reached along the SE shore, near Porto de Lomba (Figs. 1, 2). It comprises strombolian volcanic deposits and intercalated basaltic lava flows most likely erupted from a parasitic volcanic cone within the eruptive sequence of the old volcanic edifice. These deposits are covered unconformably by a polygenetic breccia several meters thick made of poorly sorted heterometric angular lava clasts cemented by a dark brown indurated matrix (Fig. 2). This unit has variable thickness and shows a channeled geometry, supporting its emplacement as a lahar deposit within an existing canyon. Notably, the upper part of the deposit grades into a much finer and lighter pyroclastic deposit with an increasing proportion of small pumices clasts revealing a significant incorporation of volcanic tephra.

The topmost part comprises almost exclusively centimetric pumice clasts and scarce lithics, with only a small proportion of muddy cement. At least three sub-layers can be distinguished, ranging in color from light grey to yellowish. These are made of alternating fine ash-fall beds and block and ash flow deposits, supporting alternating tephra sedimentation and pyroclastic flow from a volcanic ash plume. The tephra-rich horizon at the base of the topmost layer was sampled (FLO18L). While the pumice glass is partially weathered, centimetric pumice clasts contain small shiny feldspars, which were targeted for subsequent separation for single-grain ^40^Ar/^39^Ar dating. The analytical approach here adopted is particularly well suited for dating such potentially heterogeneous tephra deposits, which may incorporate inherited crystals and lithics from previous eruptions leading to a biased eruption age with bulk sample analyses. We additionally sampled a brecciated lava flow (FLO18M) directly overlying the pyroclastic deposit (Fig. 2), for subsequent K/Ar dating on separated groundmass and whole-rock geochemical analyses.

**Figure 3:**
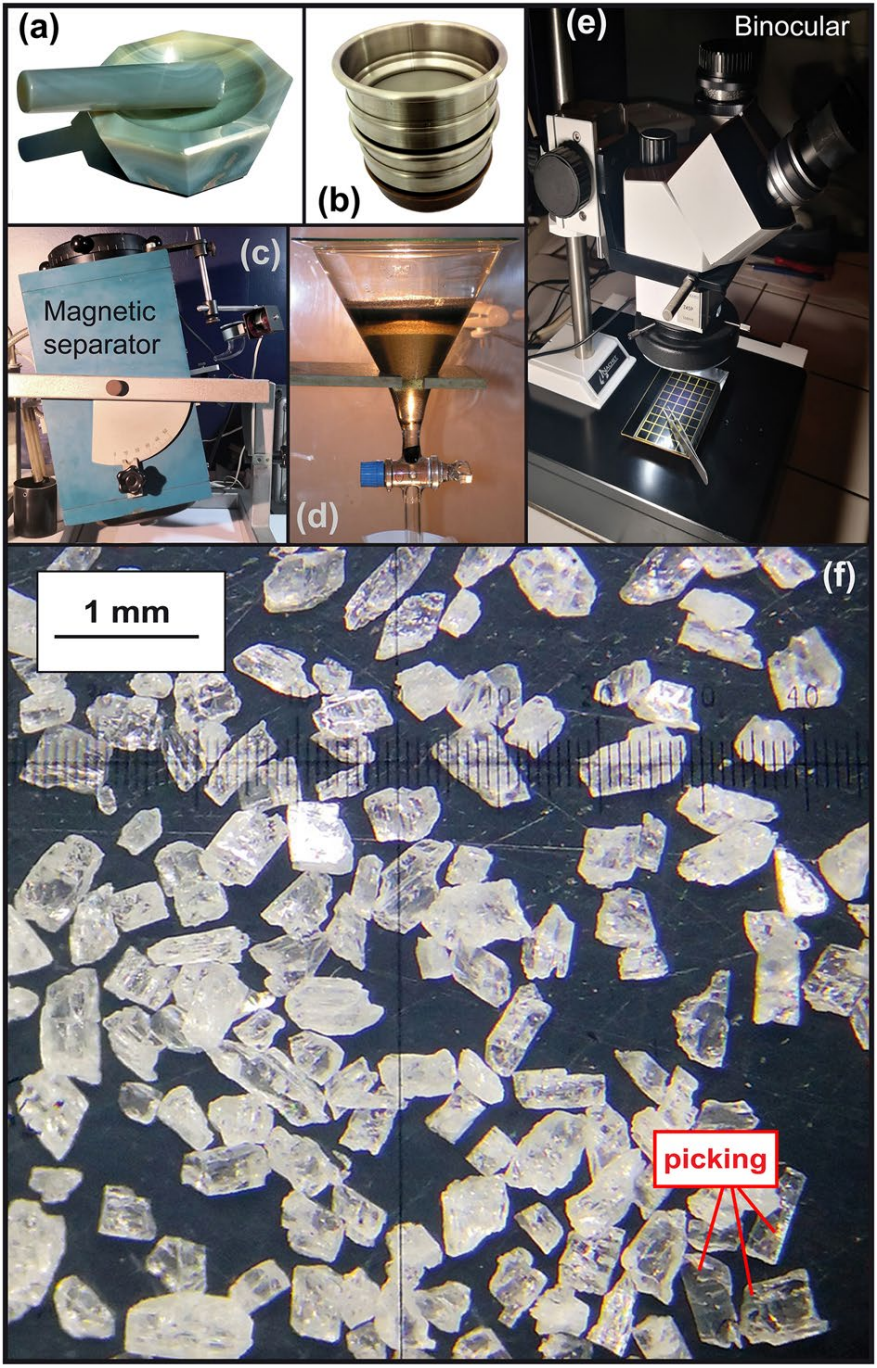
Extraction of fresh feldspars through different steps: (**a**) crushing, (**b**) sieving, (**c**) magnetic separation, (**d**) sorting by heavy liquid, (**e**) binocular observation, (**f**) picking of translucent crystals. All photos taken by Anthony Hildenbrand.

After drying in an oven at 45 °C for a few days, the pumices were carefully crushed by hand with an agate mortar and pestle to disaggregate the glass without damaging individual feldspar crystals. From the actual size of the largest observed feldspars, the sample was sieved in the range 250–500 µm, and washed with deionized water. After drying and pre-sorting with a magnetic separator, heavy liquids (Bromoform) were used to separate a homogeneous fraction of feldspars in the density range 2.57–2.59 (Fig. 3). Translucent feldspar crystals with no inclusions were then handpicked under a binocular magnifier. They were cleaned in a 5% HF solution for 3 min to remove glass selvages that remained on grains, rinsed with deionized water and dried over a few hours at moderate temperature (< 65 °C).

## Single-grain ^40^Ar/^39^Ar pumice dating

The selected crystals were sent along with Alder Creek sanidine flux monitor (ACs-2^38^) to the CLICIT facility (OSU, Oregon, USA) to be irradiated for two hours. After irradiation, twenty individual sample grains were loaded into separate cells of a copper 133 pits sample holder placed in a differential vacuum Teledyne Cetac window connected to a home-designed compact extraction line. Individual minerals were fused using a 100 W Teledyne Cetac CO_2_ laser during 15 s at a power of 2.5 W. Before being able to fuse the sample the window was backed at 110 °C for 2 days to reach ultra-high vacuum conditions. Before fusion, each crystal underwent a 10-s long sweeping at 0.3 W to remove potentially trapped unwanted gas on the crystals surface and fractures. Gases extracted during crystal fusion were firstly purified by a SAES GP 50 cold Getter for 90 s and then for 210 s by two hot SAES GP 50 Getters. Argon isotopes (i.e. ^40^Ar, ^39^Ar, ^38^Ar, ^37^Ar and ^36^Ar) were measured using a multicollector NGX 600 mass spectrometer equipped with a nine ATONA amplifier array and an electron multiplier hosted at the LSCE (PANOPLY platform, France). The simultaneous collection of all argon isotopes guarantees straightforward and very stable analytical conditions, reducing the risk of signal drift during peak switching^39^. This is especially critical for very low signals such as in our study, where the ^40^Ar signal measured on the Faraday cup is sometimes only twice the background level. Such performance is by itself not unique, as electron multipliers allow comparable detection. Nevertheless, the ATONA amplifiers further present a large dynamic range and a very linear response that is orders of magnitude better than any other Faraday cup technology based on resistance. It is particularly well suited for our moderately K-rich alkali feldspars because, soon after the return from irradiation, we were able to measure on the ATONA arrays large ^37^Ar beams that would have saturated SEM. This allowed us to correct with a great accuracy the ^36^Ar produced from ^37^Ar_Ca_ during irradiation. More technical specifications regarding the NGX 600 ATONA detector array are presented elsewhere ^40^. ^40^Ar, ^39^Ar and ^38^Ar isotopes were measured simultaneously using three ATONA amplifiers together with an electron multiplier for the ^36^Ar isotope, which was also used for the ^37^Ar in a second run. Each isotope measurement corresponds to 15 cycles of 20 s integration time. Peak intensity data were reduced using ArArCALC V2.4 ^41^. The neutron fluence J factor was calculated using co-irradiated Alder Creek sanidine (ACs) standard ACs-2 associated with an age of 1.1891 Ma ^42^ according to the K total decay constant of ^43^ (λe.c. = (0.5757 ± 0.016) × 10^–10^ a^−1^ and λβ^−^ = (4.9548 ± 0.013) × 10^–10^ a^−1^). To determine the neutron flux for each sample, at least six flux monitor crystals from pits framing the samples in each irradiation disc were used. The J-values for the sample is the following: J = 0.00056080 ± 0.00000134 (2σ). Mass discrimination was monitored overnight from measurements of 20 air pipettes with a reference ^40^Ar/^36^Ar ratio of 298.56 ^44^.

**Figure 4:**
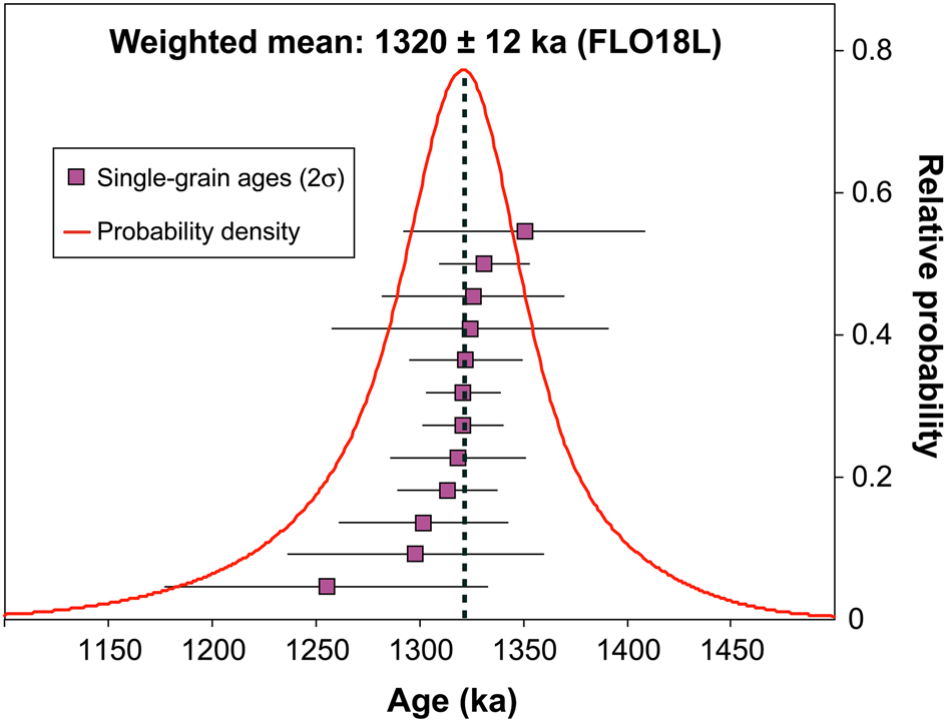
Age probability distribution and weighted mean age for the 12 grains analyzed in this study.

The analysis of only twelve feldspars was sufficient to obtain a relatively tight and statistically representative mean age, as attested by the Mean Square Weighted Deviation (MSWD = 0.6). Single-grain age determinations range between 1.255 ± 0.078 Ma and 1.351 ± 0.058 Ma (Table 1, Supplementary Data 1). The measured K/Ca ratios range between ca 4.1 ± and 7.6 (mean value around 5), indicating anorthoclase composition with variable initial K-content. Nevertheless, all individual ages are identical within uncertainties at the 2σ level supporting co-genetic crystallization of the feldspars from a relatively heterogeneous magma and strengthening the hypothesis that they all belong to a unique eruption. The inverse isochron diagram (Supplementary Data 1) is also well constrained; all data points define a well-behaved regression with a spread of 38.6% and a ^40^Ar/^36^Ar intercept value of 295.8 ± 17.0, consistent with typical atmospheric isotopic composition. The inverse isochron age is 1.322 ± 0.016 Ma. Regarding the probability density diagram, the individual data define a single Gaussian-like distribution (Fig. 4), allowing the calculation of a weighted mean age of 1.320 ± 0.012 Ma (2σ). Removing the lowest and highest individual single-grain ages would lower slightly the total uncertainty, but we prefer avoiding such artificial “trimming” ^45^. Therefore, we interpret the mean age of 1.320 ± 0.012 Ma as the age of this pyroclastic eruption.

**Table 1:** ^40^Ar/^39^Ar results on individual feldspars, and mean weighted age for pumice sample FLO18L (see full analytical details in Supplement 1). ^a^Full external error.

Analysis	^40^Ar/^39^Ar age (ka)	± 2σ (ka)	^40^Ar^a^ (%)	^39^Ar_K_ (%)	K/Ca	± 2σ
1	1322.1	27.5	81.20	7.23	4.25	0.32
2	1301.9	40.8	85.22	6.38	5.22	0.90
3	1298.0	62.0	99.47	5.29	5.17	0.99
4	1254.9	78.0	74.48	2.49	4.40	1.45
5	1318.4	32.8	85.33	6.95	4.83	0.71
6	1324.4	66.8	85.19	6.72	5.17	0.87
7	1350.5	58.2	90.01	7.58	5.28	0.89
8	1331.2	22.0	94.20	9.67	5.73	0.94
9	1313.4	24.2	86.74	13.68	7.56	1.36
10	1325.8	43.9	80.76	5.48	6.46	2.27
11	1320.7	39.3	62.87	7.50	4.07	0.41
12	1321.0	18.1	90.92	21.03	5.02	0.20
Weighted mean	1320	12^a^			4.8	0.3

## Significance of the explosive eruption

To the best of our knowledge, no pumice-bearing pyroclastic deposit with such an old age has been ever reported on Flores. The lowermost stratigraphic unit of the island referred to as the “Base Complex” comprises volcaniclastic deposits ascribed to early magma-seawater interactions during shallow submarine volcanism and island emergence ^46^. The deposit analyzed in the present study is unlikely to result from such early interactions between magma and seawater, because (1) it covers a suite of strombolian deposits and lava flows emplaced under sub-aerial conditions, (2) the channeled geometry supports downstream transport within a deep inland canyon incising an extended early sub-aerial edifice; and (3) the numerous alkali-feldspars in the pumice-rich layer here dated supports a relatively evolved magma, in contrast with the generally mafic composition of early volcanic products on Flores ^46^. Alternatively, recent trachytic fallout and pyroclastic flow deposits have been recognized on the island, but they have been erupted during the late eruptive cycles of the youngest volcano (< 0.6 Myr ^46^). Therefore, the oldest pyroclastic unit here dated cannot be linked with such recent evolution. Yet, its exact extent, volume and architecture could not be characterized, because significant erosion and dense vegetation under the wet Flores climate precluded a detailed study. Nevertheless, part of the deposit can be followed along some of the cliffs, deserving further systematic investigation. In any case, the observed association between the thick mudflow deposit and pyroclastic layers and the overall richness in pumice clasts supports a main destruction process associated with a large eruptive event at the scale of the island.

Our new ^40^Ar/^39^Ar age is undistinguishable from, but more precise and accurate than the previous K/Ar age of 1.30 ± 0.04 Ma obtained on a lava flow attributed to the late activity of the former volcano ^21^. Note that the calibrations used for K–Ar and ^40^Ar/^39^Ar are not the same but the difference involved is negligible within uncertainties (see Ref.^32^ for detailed discussion). Moreover, the age we obtained for this newly discovered pyroclastic deposit is highly consistent with the timing of the S1 flank collapse bracketed between 1.30 ± 0.04 Ma and 1.18 ± 0.09 Ma ^21^. The uniqueness of the old pumice-bearing pyroclastic eruption here recognized and its temporal coincidence with a major landslide of the island’s western flank supports a direct association between the two events. Such apparent synchronicity between large-scale flank destabilization and violent explosive eruption is comparable to the case of Tenerife Island (Canary), where the El Abrigo pyroclastic formation has been linked to sudden decompression induced by the large Icod landslide *ca* 170 ka ago (e.g., Refs.^8,47^). In a similar fashion, we interpret the pyroclastic layers in eastern Flores as likely witnessing a major explosion triggered by sudden decompression during catastrophic failure of the island's western flank at the end of the main first volcanic building stage.

## What may have triggered a large flank destabilization in Flores?

A thorough analysis of the failure mechanism(s) of Flores’ western flank is beyond the scope of the present paper. Nevertheless, the new ^40^Ar/^39^Ar age here obtained enables a qualitative discrimination of some possible causes for the triggering of the S1 landslide. Many factors favoring/enabling large-scale flank collapses on volcanic islands have been put forward over the last 25 years (e.g., Refs.^17,48^). These include ground shaking by large regional earthquakes (e.g., Ref.^49^), volcano-tectonic uplift ^24^, magma concentration along “rift-zones” ^11,12,50^ or volcano spreading over weak sediments ^51,52^. More recently, the influence of large variations in mean precipitation and/or sea level associated with global paleoclimatic changes has been also proposed (see Refs.^9,25,53^). During interglacial peaks, especially, increased rainfall infiltration may favor enhanced interactions between groundwater and magma, potentially yielding a drastic increase in pore pressure promoting failure.

In the case of Flores, a significant role for regional tectonics seems unlikely, as the island has been sitting in a relatively quiet tectonic zone (Fig. 1a). Flores has been constructed on a 10 Ma oceanic lithosphere generated by seafloor accretion at the MAR axis (e.g., Refs.^54,55^), which means that Flores sits on sediments accumulated during ca. 8 Ma. These could have worked as a soft base promoting edifice spreading and, eventually, collapse. From known half-spreading rates over the last 10 Myr, however, the Flores main volcanic edifice was already tens of kilometers away from the MAR axial Rift 1.3 Ma ago. Therefore, shallow earthquakes generated by extensional tectonics at the MAR axis fail to provide a reasonable explanation for flank disruption on Flores. In turn, significant earthquakes with magnitudes up to 6.5 frequently occur along the eastern branch of the ATJ, e.g., the Faial earthquake in 1998 ^56,57^. Nevertheless, the main shocks are located hundreds of kilometers from Flores (see Fig. 1), and thus associated ground shaking in the western Azores is negligible. We note that a structural influence of the seafloor under Flores cannot be ruled out, as the inherited fault fabric may have focused magma ascents and ultimately favor westward lateral push of the island flank. Nevertheless, such a process cannot, by itself, explain the timing of the inferred flank collapse at the end of the main volcano building-stage.

**Figure 5:**
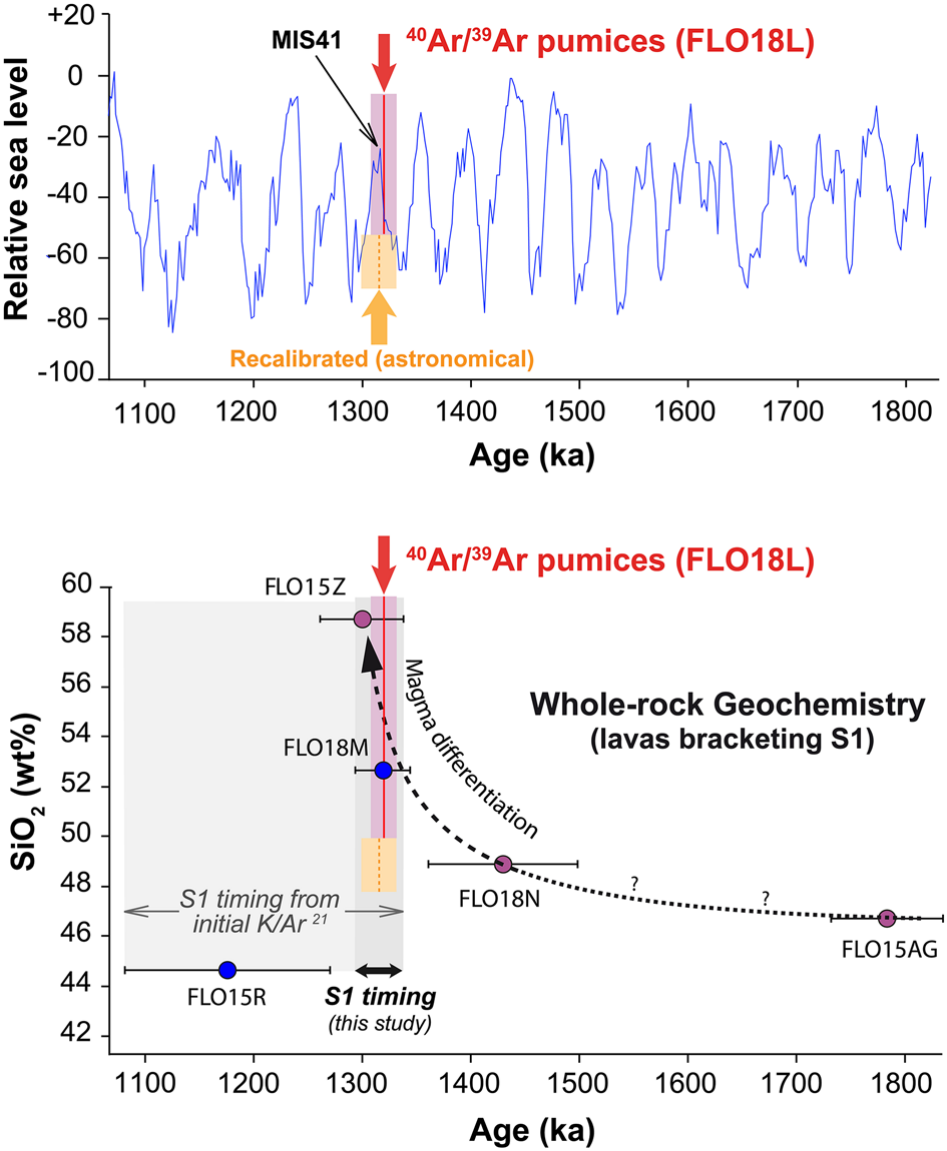
Upper panel: age of the pyroclastic event with respect to global sea level variations ^58^. Relative sea level is indicated with respect to present; MIS41 after ^59^. Lower panel: SiO_2_ vs age diagram showing the geochemical evolution of selected lavas bracketing the S1 flank collapse. The new ^40^Ar/^39^Ar age of the pyroclastic event supports triggering of the pyroclastic event at the end of a main differentiation cycle.

Investigating the influence of paleoclimatic changes requires age recalibration, as global variations recorded by δ^18^O in marine records ^58^ are tied to astronomical tuning. This means considering a reference value of 1.1851 ± 0.0004 Ma for ACs ^45^, which introduces a difference of 0.34%. In our case, however, the difference is minor, as the weighted mean age for our sample FLO18L becomes 1.315 ± 0.016 Ma. Plotting the revised age for FLO18L against global sea level variations ^59^ shows that the pyroclastic event occurred near Marine Isotopic Stage 41 (Fig. 5). The age uncertainty, however, comprises both the rapid glacial–interglacial (G–I) transition preceding the MIS41 peak and the onset of the sub-glacial period between MIS41 and MIS39. It is worth noticing that the Marine Isotopic Stage age uncertainties on the astronomical tuning method are very hard to evaluate and often neglected. However, they probably exceed 5 ka (see discussion in Ref.^58^) for the period older than 500 ka. Recent paleoclimatic reconstructions from well-dated paleosols spanning the late Quaternary in the central and eastern Azores have shown that the largest global climatic transitions recorded major shifts in mean air temperature and precipitation ^32,60^. For instance, mean rainfall increase by up to 50% occurred during the MIS11 transition about 425 ka ago, with an associated sea level rise of at least 120 m (Supplementary Fig. 1). In comparison, the maximal amplitude associated with MIS41 transition about 1.32 Ma ago was relatively modest, with eustatic sea level variations not exceeding 40 m. Associated changes in air moisture and rainfall regime may thus have been rather limited at the time. Consequently, paleoclimatic changes near MIS41 seem unlikely to have been the main trigger for the S1 lateral collapse in Flores, and additional factors such as magmatic processes should be here considered.

New whole-rock (WR) geochemical analyses provide additional (qualitative) insights into the potential role of magmatic processes. Unfortunately, the pumices themselves could not be used because the glass is too weathered. Alternatively, we targeted reasonably fresh lava flow samples bracketing the S1 landslide. Samples from the pre-collapse volcanic succession include the oldest and youngest lava flows attributed to the early and late activity of the older volcano and dated between 1.78 ± 0.05 Ma (sample FLO15AG) and 1.30 ± 0.04 Ma (FLO15Z), respectively^21^. We added an intermediate lava flow (FLO18N) from the lava succession covered in unconformity by the pyroclastic deposit (location in Fig. 1c). New K/Ar analyses on a groundmass separate of this sample in GEOPS yielded a mean age of 1.43 ± 0.07 Ma (Table 2). Full details on sample preparation, unspiked K/Ar analytical procedure and age calculation can be found elsewhere (e.g., Refs.^21,32,61^). A new K/Ar age of 1.32 ± 0.02 Ma was further obtained on the groundmass of our lava flow sample FLO18M (Table 2). Within uncertainties, the latter age is fully consistent with our new ^40^Ar/^39^Ar mean age of 1.32 ± 0.01 Ma for the explosion, and with the age of 1.30 ± 0.04 Ma measured on the pre-collapse lava sample FLO15Z ^21^. Note, however, that the lava flow FLO18M directly sits onto the pyroclastic deposit (lack of paleosol or unconformity) and is therefore interpreted as erupted soon after the collapse. The whole-rock analysis on this sample may thus give hints on the composition of the magma that erupted shortly after the explosive event. Finally, a new whole-rock geochemical analysis on a basic lava flow from the early post-collapse activity (sample FLO15R) dated at 1.18 ± 0.09 Ma by ^21^ was made to constrain potential evolution in magma chemistry within a few tens of kyr after the S1 collapse.

**Table 2:** New K/Ar dating on separated groundmass (125–250 µm) of our lava flow samples FLO18M and FLO18N. The uncertainty on each determination is obtained as the quadratic sum of all independent sources of uncertainty, i.e. uncertainty on the K content (1%), uncertainty on the calibration of the mass spectrometer (1%), and the uncertainty on the amount of radiogenic argon. The mean age (^a^) is calculated by weighing by the inverse of the variance. Full details on analytical procedure, decay constants and calibration can be found elsewhere (e.g., Refs.^21,32,61^). Sample coordinates in decimal degrees. *Lat.* Latitude, *Long.* Longitude, *Alt.* Altitude.

Sample	Lat. (N)	Long. (W)	Alt. (m)	K%	^40^Ar^a^ (%)	^40^Ar^a^ (10^12^ at/g)	Age (Ma)	± 2σ (Ma)
FLO18N	39.4141319	31.1503361	2	1.697	2.2	2.509	1.415	0.135
					2.1	2.519	1.420	0.141
					2.2	2.605	1.469	0.141
					2.0	2.517	1.420	0.148
						Mean^a^	1.431	0.070
FLO18M	39.4147305	31.1500082	5	2.345	13.7	3.228	1.317	0.042
					12.1	3.235	1.320	0.043
					11.7	3.256	1.329	0.044
						Mean^a^	1.322	0.025

The analyzed lavas show significant compositional variations as a function of time (Table 3, Fig. 5, Supplementary Fig. 2). The incompatible elements content increases during the late activity of the former shield, whereas the post-collapse activity shows a sudden drop. Conversely, MgO contents display an anti-correlated behavior further supporting differentiation through crystal fractionation of olivine and clinopyroxene in a magma reservoir. Strikingly, the lava flow FLO18M here attributed to the immediate post-collapse activity shows intermediate major elements composition. Selected trace elements provide additional insights. Nb and Zr have comparable behavior with respect to magma crystallization. As such, Zr/Nb ratios are expected to show constant values within a given magmatic series, whereas significant Zr/Nb variations support changes in melting conditions and/or source composition (e.g., Refs.^62,63^). In the present case, Zr/Nb shows no significant variations for the lavas erupted before the main S1 collapse (Supplementary Fig. 2). We are aware that such a large period may not reflect a single eruptive cycle, and that only three samples are not enough to constrain a detailed evolution of the magmatic system. However, the very reproducible Zr/Nb values support overall similar conditions of melt production during the bulk construction of the main older volcano. Interestingly, the similar Zr/Nb ratio obtained on our sample FLO18M dated at 1.32 ± 0.02 Ma suggests the persistence of the main magmatic signature within a very short period of time following the landslide. In contrast, distinct Zr/Nb for the basaltic post-collapse lava flow FLO15R supports a change in the conditions of magma generation during the few tens of kyr following the main destabilization event, as documented on many other volcanic ocean islands (e.g., Refs.^11,64^).

**Table 3:** Whole-rock geochemical analyses on lava flow samples bracketing S1 flank collapse. Major and trace elements measured at the SARM national facility (Nancy) by ICP-OES and ICP-MS, respectively. ^a^K/Ar ages from Ref.^21^ with uncertainties quoted at 2σ; ^b^K/Ar age on samples FLO18M and FLO18N (this study).

Sample	FLO15AG	FLO18N	FLO15Z	FLO18M	FLO15R
Age (Ma)	1.78^a^	1.43^b^	1.30^a^	1.32^b^	1.18^a^
2σ uncertainty (Ma)	0.05^a^	0.07^b^	0.04^a^	0.02^a^	0.09^a^
Major element oxides (wt%)					
SiO_2_	46.70	48.95	58.74	52.63	44.62
TiO_2_	2.89	2.78	1.00	2.07	3.12
Al_2_O_3_	15.84	15.90	17.57	17.49	15.10
Fe_2_O_3_^a^	10.56	10.73	6.76	7.38	12.45
MnO	0.19	0.58	0.26	0.28	0.21
MgO	4.69	2.46	1.20	1.84	4.14
CaO	9.47	7.41	2.75	6.80	10.34
Na_2_O	3.51	4.27	7.07	5.84	3.13
K_2_O	2.13	2.14	3.75	2.63	1.72
P_2_O_5_	0.72	0.90	0.29	0.78	0.69
Loss on ignition (LOI)	2.53	3.69	1.02	2.55	3.62
Total	99.24	99.81	100.41	100.28	99.11
Selected trace elements (ppm)					
Zr	237	201	347	242	210
Nb	71.7	60.9	105	75.6	75.8

Our restricted dataset allows us to discuss potential links between magma dynamics and edifice destabilization. The general pattern for the old lavas, although based on a limited number of samples, supports a relatively simple process of magma evolution ending with late differentiation in a magma reservoir. From high-resolution geochronological and geochronological studies on other oceanic islands, such differentiation patterns typically occur over timescales of only a few ka to tens of ka (e.g., Refs.^8,11,15^). On Flores, the trachytic lava sample FLO15Z thus may not represent the ultimate product of differentiation, and even more evolved magma composition may have been reached just before the S1 collapse. Experimental works have shown that highly evolved alkaline lavas from intra-plate settings reach viscosities similar to some andesites and dacites erupted in continental-arc settings, sometimes leading to comparable eruptive behavior ^65,66^. In the case of Mount St. Helens (MSH, USA) in May 1980, intrusion of a viscous dacitic magma body within a mature (unstable) volcano cumulatively produced deformation by several tens of meters, favoring flank failure and large debris-avalanche run-out, followed by subsequent lateral blast and development of a Plinian eruption ^67,68^. Within a few years after the MSH 1980 event, a relatively degased magma was further erupted onto the landslide scar.

**Figure 6:**
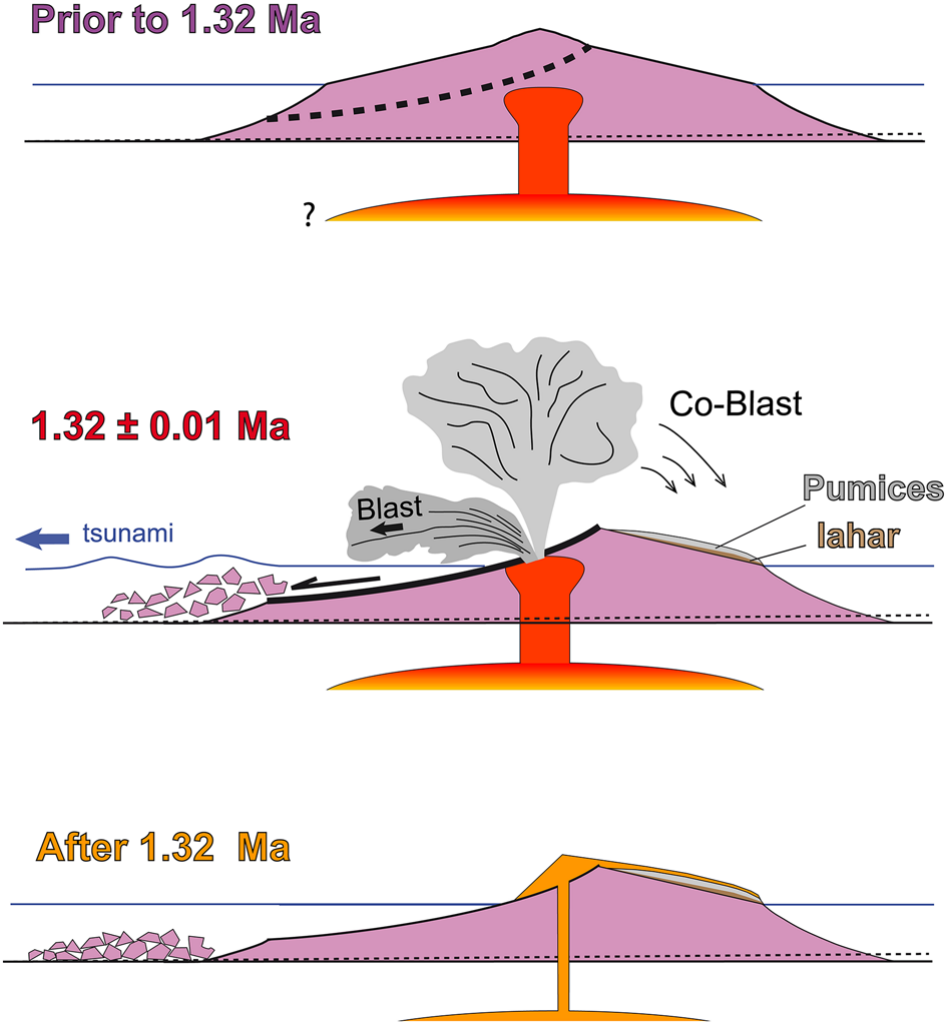
Schematic model linking magma differentiation at depth, viscous magma intrusion, edifice inflation, catastrophic failure of the western island flank, development of a blast and eruptive column, followed by late degased lava eruption (exact vent location unknown).

A similar scenario of course could also be advocated for Flores (Fig. 6). Viscous magma intrusion into an edifice sitting on soft sediments on the western MAR flank seems like a plausible conditions to favor flank spreading and westward lateral collapse. Notably, grading of the lahar deposit into pyroclastic deposits supports a genetic link between mudflow and the explosive eruption. We interpret this as evidence for ground shaking during failure of the western island flank, directly followed by a decompression-induced blast and subsequent development of a main eruptive column (Fig. 6). The variable Ca/K measured for the distinct feldspars in the pumice level (FLO18L) suggest that the pyroclastic eruption was indeed fed by a somehow heterogeneous magma batch, e.g., suddenly removed from a zoned magma chamber. Such a possibility is further supported by the intermediary composition of the late brecciated lava flow FLO18M, which suggests the late eruption of a (denser) magma that was left over from the explosive eruption.

In summary, we now build a qualitative scenario based on the available information we have gathered: the main blast likely occurred towards the west, in response to major collapse unloading. The eastern rather small-sized deposit we dated at 1.32 ± 0.01 Ma is probably the only onshore accessible witness of a much larger volume of material erupted during this event. Notably, the homogeneous ages obtained on the several grains from the upper layer here analyzed suggest a juvenile character of the pumices, providing a proximal sampling of the eruptive column developed subsequent to an initial lateral blast. Following the terminology adopted by ^68^ after recent re-analyses of the deposits generated by the Mount St. Helens eruption in May 1980, the deposit we dated at 1.32 ± 0.01 Ma is here interpreted as a “co-blast” deposit.

## Conclusions

Single-crystal ^40^Ar/^39^Ar dating of individual feldspar grains from a pyroclastic deposit preserved on the eastern flank of Flores Island (Azores) yielded a robust weighted mean age of 1.32 ± 0.01 Ma. From geological, geochronological and geochemical evidence, we propose that this unique and likely voluminous differentiated explosive eruption provides the first and direct clues on the timing of a major sector collapse, which removed the whole western side of the former island. We currently favor a scenario where volcano/magmatic dynamics is the main driving factor of this flank instability, possibly aided by the presence of soft sediments accumulated on the western flank of the MAR prior to island’s edification. The event marks the end of the main phase of growth of the former shield volcano, and was possibly triggered by intrusion of a viscous and volatile-enriched magma batch following differentiation in a crustal reservoir. We hereafter speculate upon possible processes, and propose a first scenario where the ascent and accumulation of a significant volume of such relatively felsic magma possibly undergoing volatile exsolution at a shallow level may have caused edifice inflation. This ground deformation (± another external mechanism) may have initiated flank collapse, and the depressurization resulted in a major explosive eruption. We think that the single-grain ^40^Ar/^39^Ar dating approach developed in this work could be further used to establish a robust history of pyroclastic eruptions eventually associated with other flank collapses in Flores and elsewhere. In the Azores, especially, many large landslides have been recognized ^18,19,69–74^, but most of them remain poorly dated. Further evaluation may then be of high added value for regional correlations through complementary geochemical investigations, e.g., by systematic analyses of radiogenic isotopic signatures (insensitive to weathering). As most of the (Plinian?) material may have been accumulated around Flores, it may constitute a remarkable tephra layer tie point of potential high interest for tephrochronological studies in marine sediment records around the archipelago.

Overall, our study shows that high-sensitivity mass spectrometers have reached analytical performances allowing to measure precise ages on relatively small and moderately K-rich single feldspars, which is particularly suitable for meaningful dating of potentially heterogeneous blasts and tephra deposits induced by large-scale flank collapses during the late Quaternary.

### Supplementary Information

Supplementary Information 1.

Supplementary Information 2.

## Data Availability

All data presented in the paper are available upon request to the corresponding author.
