# Supplementary material for: Precise dating of large flank collapses by single-grain 40Ar/39Ar on pyroclastic deposits from the example of Flores Island (Azores)
Source: Sci Rep. 2024 May 24;14:11905. doi: 10.1038/s41598-024-62583-1 (PMC11126614; doi:10.1038/s41598-024-62583-1)
Supplement: Supplementary file 2 — Supplementary Information 2. [file 41598_2024_62583_MOESM2_ESM.docx]

**Supplementary figures** for the article “Precise dating of large flank collapses by single-grain ^40^Ar/^39^Ar on pyroclastic deposits, from the example of Flores Island (Azores)” by Hildenbrand and co-authors

**Supplementary Figure 1:** Marine Isotopic Stages (MIS) and sea level variations over the last 2 Myr [58, 59]. The various MIS are indicated in bold.

**
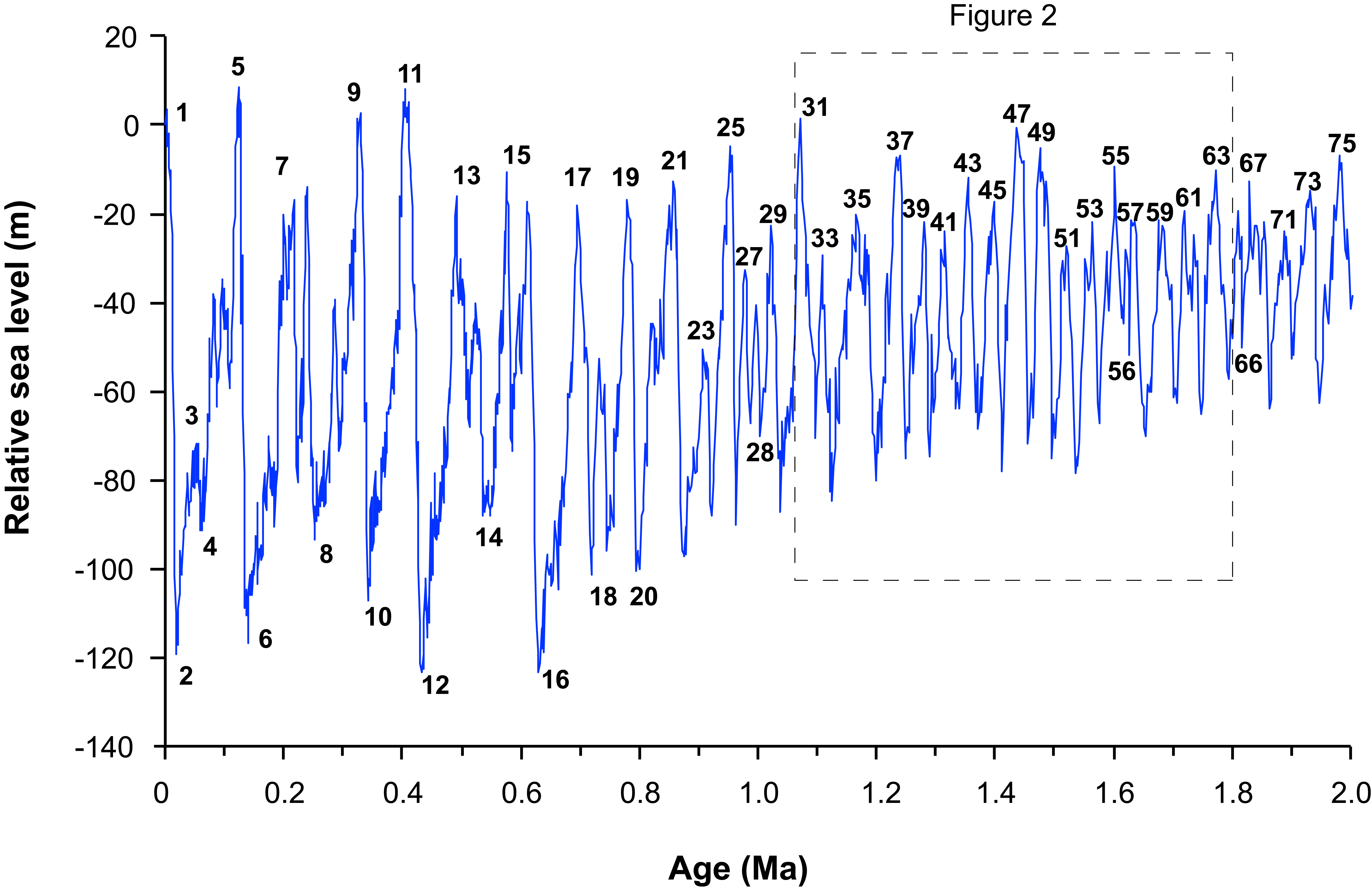
**

**Supplementary Figure 2:** Variations in major and trace element composition as a function of age for lavas bracketing the S1 slide. Pre- and post-collapse lava flows are shown with purple and blue colors, respectively.

**
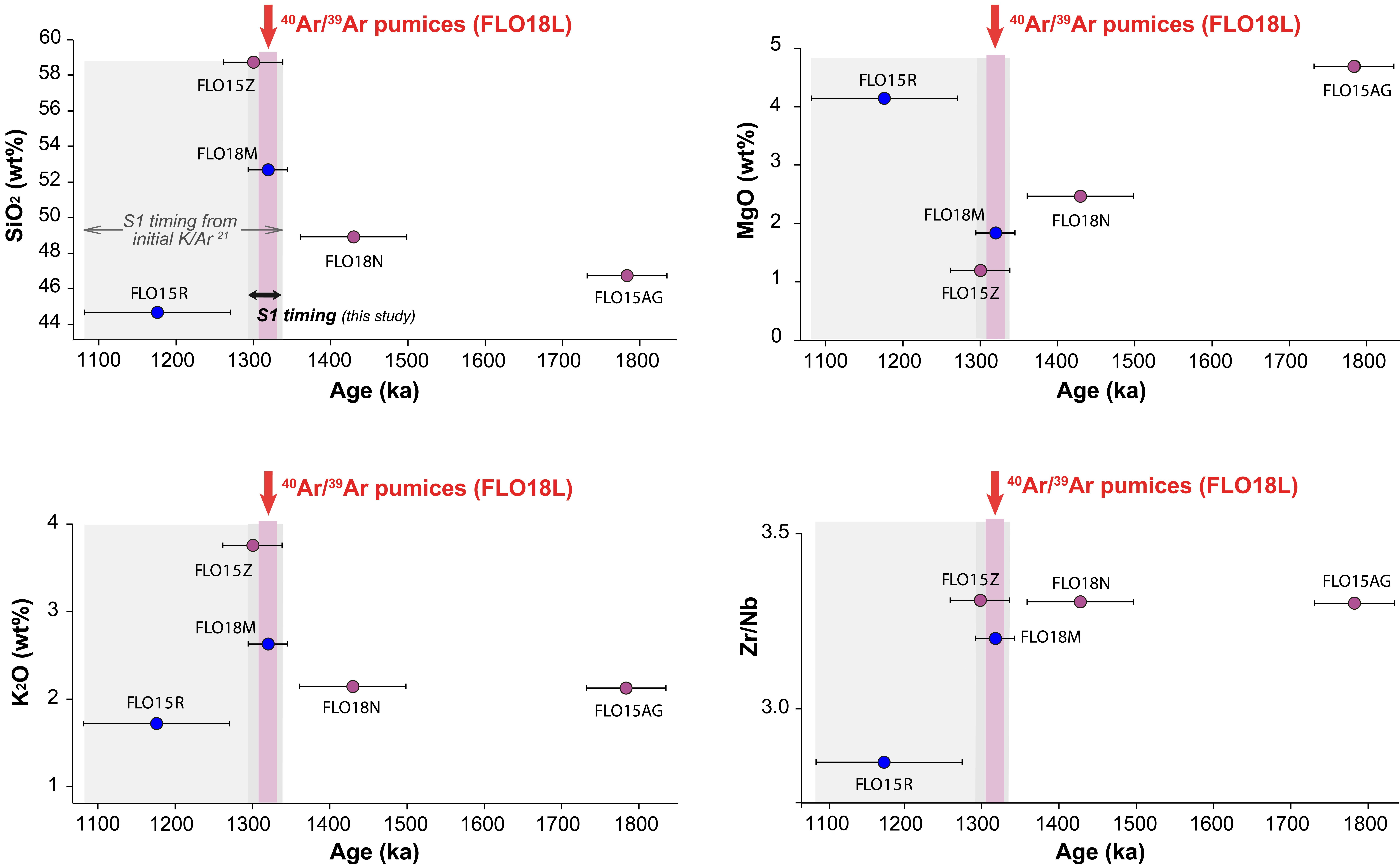
**
